# I Need a CAVAA: How Conversational Agent Voting Advice Applications (CAVAAs) Affect Users' Political Knowledge and Tool Experience

**DOI:** 10.3389/frai.2022.835505

**Published:** 2022-05-12

**Authors:** Naomi Kamoen, Christine Liebrecht

**Affiliations:** Department of Communication and Cognition, Tilburg School of Humanities and Digital Sciences, Tilburg University, Tilburg, Netherlands

**Keywords:** Voting Advice Applications, Conversational Agents, chatbot design, usefulness, ease of use, playfulness, voting intention

## Abstract

In election times, millions of voters consult Voting Advice Applications (VAAs) to learn more about political parties and their standpoints. While VAAs have been shown to enhance political knowledge and increase electoral turnout, research also demonstrates that voters frequently experience comprehension problems when responding to the political attitude statements in a VAA. We describe two studies in which we test a new type of VAA, called Conversational Agent VAA (CAVAA), in which users can easily access relevant information about the political issues in the VAA statements by asking questions to a chatbot. Study 1 reports about an online experiment (*N* = 229) with a 2 (Type: traditional VAA/CAVAA) x 2 (Political sophistication: low/high) design. Results show that CAVAA users report higher perceived political knowledge scores and also answer more factual knowledge questions correctly than users of a regular VAA. Also, participants' CAVAA experience was evaluated better. In Study 2 (*N* = 180), we compared three CAVAA designs (a structured design with buttons, a non-structured design with an open text field, and a semi-structured design with both buttons and an open text field), again for higher and lower politically sophisticated users. While the three designs score equally high on factual and perceived knowledge indicators, the experience of the structured CAVAA was evaluated more positively than the non-structured version. To explore the possible cause for these results, we conducted an additional qualitative content analysis on 90 chatbot-conversations (30 per chatbot version). This analysis shows that users more frequently access additional information in a structured design than in a non-structured design, whereas the number of break-offs is the same. This suggests that the structured design delivers the best experience, because it provides the best trigger to ask questions to the chatbot.

## Introduction

In many European countries, there is an increasing number of political parties competing for the voter's favor. During the national elections of 2021 in the Netherlands, for example, voters had no less than 37 political parties to choose from, of which 20 participated for the first time. Both the large numbers of parties and the influx of new parties are important reasons why citizens in the Netherlands, and in other multi-party democracies too, find it hard to make a well-informed vote choice (Garzia and Marschall, 2012; Kamoen et al., 2015). A lack of political knowledge, in turn, counts as one of the most important reasons to abstain from voting (Delli Carpini and Keeter, 1996).

Parallel to the growing complexity of political landscapes runs the emergence of Voting Advice Applications (VAAs), like the Dutch *Stemwijzer*, the German *Wahl-O-Mat*, and the Swiss *Smartvote*. These online tools (also see De Graaf, 2010) provide users with a voting advice based on their answers to approximately 30 attitude statements about political issues, such as “Dog tax should be increased.” This formula seems to work well: during the 2021 Dutch national elections, for example, the two most popular VAAs in the Netherlands were consulted more than 10 million times (NRC Handelsblad, 2021), and also in other countries usage figures are both high and on the rise (Garzia and Marschall, 2012; Marschall, 2014).

Not only are VAAs popular, they have also been shown to positively impact several aspects of democratic citizenship, as they enhance users' (perceived) political knowledge (Schultze, 2014) and (therefore) also elevate the turnout at the elections (Garzia et al., 2014; Gemenis and Rosema, 2014). However, despite their demonstrable positive effects, there is also research showing that VAA users frequently experience comprehension problems when answering VAA statements; on average, one in every five VAA statements causes comprehension problems (Kamoen and Holleman, 2017). This is because, for one, users lack semantic knowledge about the meaning of political terms (e.g., “What is dog tax?”), and second, because they lack background knowledge about the political issue at stake (e.g., “How high is the dog tax?”; cf. Kamoen and Holleman, 2017).

Current VAAs usually do not provide much information along with the VAA statements, and therefore one might expect that VAA users would make an effort themselves to consult a search engine on the web so as to solve their comprehension problems. This expectation, however, is not met (Kamoen and Holleman, 2017). Instead of making an effort, users have been shown to display behavior referred to in the survey literature as “satisficing behavior” (cf. Krosnick, 1991), which means that they make only a very minimal effort to provide an answer that is more or less suitable. This behavior is exemplified by VAA users making assumptions about the question meaning (see Kamoen and Holleman, 2017), and by disproportionally selecting a neutral or no-opinion response option (see Baka et al., 2012; Van Outersterp et al., 2016). Obviously, this has repercussions for the validity of the voting advice, which is based on the user's answers. Moreover, it indirectly also affects the actual vote casted, as VAA users have been shown to take the VAA's recommendations into account when deciding which political party to vote for (Andreadis and Wall, 2014; Wall et al., 2014).

In light of the above, a relevant question is how VAAs can offer additional information along with the VAA statements in a way that matches with this processing mode of low elaboration. Some researchers (Terán and Drobnjak, 2013) have put forward the idea that it could be beneficial to implement a synchronous form of communication in VAAs. In line with that idea, the current article investigates whether the implementation of a real-time Conversational Agent (chatbot) into a VAA is a good way to provide users with additional information in this specific usage context. In a VAA designed as a chatbot, users can request additional information about the political attitude statements. The fact that this information can be requested “on demand”, makes the implementation of a chatbot-functionality a promising way of information provision. This is because in a chatbot users only have to make a minimal effort to obtain the information, which responds to the processing mode users are in [Baka et al. (2012), Van Outersterp et al. (2016), and Kamoen and Holleman (2017)]. We will refer to this new application as a CAVAA, which is an abbreviation for Conversational Agent Voting Advice Application.

In the present manuscript, we will first discuss relevant literature on Conversational Agents, and we will use this literature to formulate hypotheses about how a CAVAA could affect both political measures and user experience measures. We then report about two experimental studies on CAVAAs. In the first study, we experimentally compare a CAVAA with a regular VAA on both political knowledge measures and a broad tool evaluation measure. In a second study, we subsequently compare three ways in which a CAVAA can be designed: structured with buttons, non-structured with an open chatbot functionality, and semi-structured with both buttons and an open chat functionality. We compare these three versions again on political knowledge indicators and tool evaluation measures. In both Study 1 and Study 2, we explicitly make a distinction between different user types — those with lower and higher levels of political sophistication — to explore if any of the effects are different for these two groups with different capacities and information needs.

### Conversational Agents

Conversational Agents (CAs) are interactive dialogue systems that enable humans to engage in real-time conversations by asking specific questions to the system and receive an answer in return. They exist in different types. For one, CAs may be embodied or disembodied, meaning that they may either have a (virtual) body or face that allows both verbal and non-verbal communication, or are merely presented in a message-based interface without a virtual image (Krämer et al., 2009). Second, they exist in different modalities, as some CAs are text-based and others voice-based (Gnewuch et al., 2017). Third, CAs can be developed to either respond to all of the user's messages regardless their topic, or they can be task-oriented replying only to questions within a specific domain (Gnewuch et al., 2017).

The current study focuses on a disembodied, text-based CA that is task-oriented and only responds to comprehension questions about political statements in a VAA. While there are some studies that have examined CAs for other political purposes, for example to intervene in social media discussions about politics (Forelle et al., 2015; Suárez-Serrato et al., 2016), research is lacking on task-oriented CAs to which users can ask questions about political topics. Similar to task-oriented systems in other domains, the main aim of a CAVAA is to help users achieving their goal (Yan et al., 2016; Wu et al., 2018), which is in this case: obtaining knowledge about current political issues in a low-threshold way that ultimately helps deciding which political party to vote for.

There are various reasons to believe that integrating a task-oriented CA into a VAA is feasible and beneficial. In current traditional VAAs, users have been found to frequently experience two specific types of comprehension problems when answering the statements: semantic problems (users lacking knowledge about the meaning of a term used in the question) and pragmatic meaning problems (users lacking knowledge about the current state of affairs with respect to the political issue; see Kamoen and Holleman, 2017). One advantage of integrating a task-oriented CA, is that this tool can well be trained to answer these specific and predictable user questions. In other words, the context is demarked and the types of comprehension problems VAA users face are known, which makes it possible to develop a task-oriented system. A second benefit of providing information in a CA, is that users do not have to switch between channels (e.g., the VAA and a search engine) to get an answer to their question. This is an advantage, as research demonstrates that VAA users are in a processing mode of low elaboration; they hardly make an effort to look up information on the web via search engines to solve these comprehension problems (this happens in only 1.4% of the cases, see Kamoen and Holleman, 2017). Studies in the field of survey research demonstrate that the lesser effort respondents have to make to access information, the more likely they are to do so (Galesic et al., 2008). Therefore, allowing users to find information on the same page is an important advantage. What is more, while general search engines like Google provide information that is not structured to meet the users' specific information need, a CAVAA can provide the user with tailored information. Hence, a CA can provide information that really responds to the user's needs. Finally, the conversational nature of CAs enables users to ask multiple questions about one political statement (e.g., to look up both semantic and pragmatic information), while at the same time users who do not require any additional information are not confronted with extra ballast. All in all, CAs can offer tailored information in a way that matches with the users' processing mode of low elaboration.

In light of the above, it can be expected that a task-oriented CAVAA can outperform a traditional VAA without additional information on both political measures and user experience measures. As a CAVAA provides information in an easily accessible way, we expect this tool to increase the user's perceived and factual political knowledge more than a traditional VAA. Moreover, as there is a strong relation between a citizen's knowledge and political self-confidence, and the chance of going voting (Delli Carpini and Keeter, 1996), we also expect CAVAA users to report more frequently than VAA users that they have the intention to vote in the upcoming elections. Finally, the CAVAA's user experience may be better, as the main reasons for using a task-oriented CA (see Brandtzaeg and Følstad, 2017) match with the main reasons for using a VAA (see Van de Pol et al., 2014); both VAAs and task-oriented CAs are used for productivity and for fun. As a CAVAA therefore probably better assist users in obtaining their goal of finding political information in a fun way, we also expect the user experience of this tool to be evaluated better.

### Political Sophistication

When developing a CA for the domain of VAAs, a relevant fact is that VAAs are normally used by very different kinds of users (e.g., Van de Pol et al., 2014); while a slight majority of VAA users is highly educated and has some political interest and knowledge before entering the tool, about 30 to 40 percent of the VAA users are not highly educated, not much interested in politics and not highly knowledgeable. This latter group of VAA users is both most likely to experience comprehension problems when answering the VAA statements (see Kamoen and Holleman, 2017), and most likely to expire in a mode of low elaboration, as the chance of showing low elaboration processing behavior depends on (a) the difficulty of the task (b) the motivation to engage in the task and (c) the cognitive capacities (Krosnick, 2000). In the current research, we will therefore investigate the effects of VAAs vs. CAVAAs for different groups of users, making a distinction in two groups based on the user's political sophistication.

The concept of political sophistication has been described by Luskin (1990) as the combination of a person's cognitive ability to understand information (usually measured with level of education), the extent to which one is already informed (in a political context usually measured as general prior political knowledge), and the motivation to put effort into the collection of more information (usually measured as political interest). Although not all previous studies focusing on political sophistication have actually used an aggregate index of all these three different aspects (but rather a subset; e.g., see Holleman et al., 2016), we will include all three parameters to make a distinction between higher and lower politically sophisticated users. This way, we aim to do some right to the heterogeneity of voters who consult a VAA in real life.

There are two scenarios for how the political sophistication of users may moderate the effect of VAAs vs. CAVAAs. On the one hand, the effects as they were hypothesized earlier may be stronger for those users with lower levels of political sophistication, as they will experience most comprehension difficulties when answering the VAA statements and therefore have more potential to learn (see Kamoen and Holleman, 2017 who made a similar suggestion). On the other hand, it is probably also users with a lower level of political sophistication who have more difficulties recognizing their comprehension problems, and, if they do recognize these, to phrase a question to resolve these problems (Elling et al., 2012). Therefore, it is also possible that they will benefit to a lesser extent from a CAVAA compared to users with a higher level of political sophistication. As we are uncertain about which line of reasoning to follow here, but at the same time want to do right to any possible differences between these two important user groups, we will explore the moderating effect of the users' level of political sophistication.

## Study 1: Method

### Design

To test the hypotheses, an online experiment has been conducted with a 2 (type of VAA: traditional VAA or CAVAA) x 2 (political sophistication: low or high) between-subjects design.^1^ Each participant has been randomly assigned to one of the VAA conditions; political sophistication has been operationalized as a quasi-experimental variable. The design of the study was approved by the Research Ethics and Data Management Committee of Tilburg University, identification code: REDC #2020/060.

### Materials

#### (CA)VAA Content

The core content of both the traditional VAA and the CAVAA is formed by a set of 20 political attitude statements to which users were asked to express their opinion on a three-point scale (Agree – Neutral – Disagree), supplemented with an additional fourth don't know-option. A total of 17 out of the 20 statements were taken from existing VAAs of the Dutch brands *Stemwijzer* and *Kieskompas*, as they were used in the VAAs developed for the Dutch national elections of 2017. We selected only those statements that were (a) still relevant at time of administration of the experiment and (b) that were expected to lead to semantic and pragmatic comprehension problems (Kamoen and Holleman, 2017). In addition, we formulated three statements ourselves so that the total number of VAA statements is 20 (which is about the minimum number of statements in a naturalistic VAA, see Van Camp et al., 2014). These three new statements were about the Corona-crisis, which was the topic that dominated the political debate at the time of administration.

#### Traditional VAA

The traditional VAA was constructed in the survey program Qualtrics.^2^ In this tool, statements were offered to the respondents one-by-one. After having answered the 20 statements, the tool provided the user with a voting advice. This voting advice was based on the number of matches between the users' answers and political party's issue positions. We used the political party's websites to code the party positions on the statements. The voting advice was depicted as a percentage of agreement with a set of eight political parties (the largest parties in the polls at time of administration), comparable to how the voting advice is displayed in *Stemwijzer*.

#### CAVAA

The CAVAA was developed using software of the company Flow.ai.^3^ Flow.ai is a platform that allows creating CAs for websites and social media channels. We developed a semi-structured CA in which users could access additional information in two ways: (1) by clicking on information buttons and (2) by typing a question in an open chat window.

**Figure 1:**
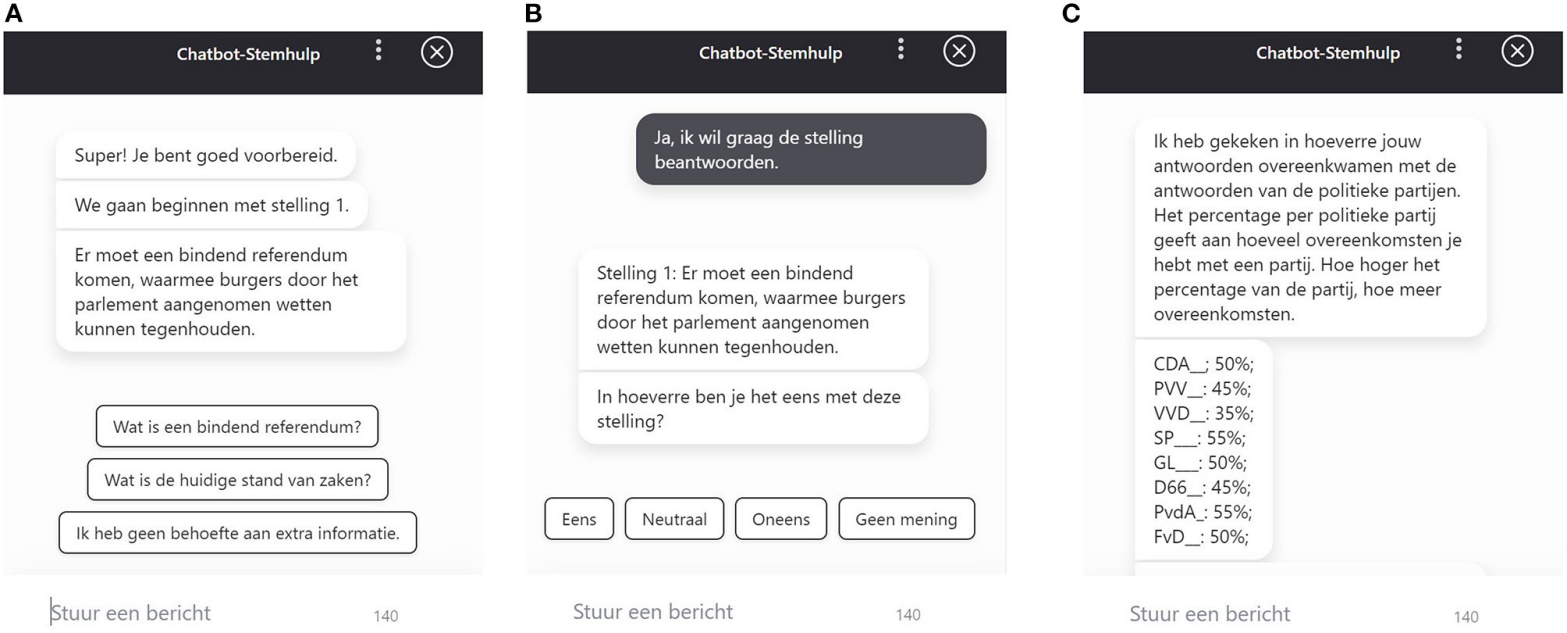
**(A)** Example of a CAVAA statement (*We will start with statement 1. There should be a binding referendum with which citizens can stop laws being implemented*), three buttons (*What is a binding referendum, What is the current state of affairs*, and *I do not need additional information*), and a text-entry functionality to request specific information. **(B)** Example of a CAVAA statement and response options (*There should be a binding referendum with which citizens can stop laws being implemented*. *To what extent do you agree with this statement? Agree, Neutral, Disagree, No Opinion*). **(C)** Example of the CAVAA voting advice (*I have checked to what extent your answers match with the answers of political parties. Below you can find the percentage of agreement with each political party. The higher the percentage, the more similar answers you have provided as the political party mentioned*).

Similar to the majority of task-oriented information retrieval CAs in other contexts (Sethi, 2020), the CAVAA developed for the purposes of the current research was designed as a rule-based system in which conversations are mapped out like a flow chart that guides users through a decision tree (the architectural design and conversational flow can be found in Dataverse, https://doi.org/10.34894/J0CM5K). In the CAVAA, information buttons (see Figures 1A–C) guide users through the tool and enable users to access two types of information: (1) information about a difficult concept in the attitude statement (semantic information) and (2) information about the current state of affairs with respect to the topic in the statement (pragmatic information). The choice to add these specific information buttons was made because the study by Kamoen and Holleman (2017) indicates that these are the types of comprehension problems VAA users most frequently encounter.

The button that could be used to access the semantic information always read *What is X?*, where X was the specific term in the statement that was explained (e.g., *What is a binding referendum?*). Clicking this button led to a short explanation of the term (e.g., “A referendum is a popular vote about a political issue. In case of a binding referendum, the government should follow the outcome of this popular vote”). The button users could click on to access pragmatic information always had the label *What is the current state of affairs?*. Clicking this button always led to more information about the current state of affairs (e.g., “Between July 2015 and July 2018 it was possible for Dutch citizens to request an advisory referendum for certain treaties. In July 2018, the law allowing an advisory referendum has been repealed. The law for allowing an advisory referendum should be reinstalled first to allow other referenda such as a binding referendum”). The information in the CAVAA responses was always based on reliable resources, such as government websites or online dictionaries (the original experimental materials and an English example of the stimulus materials can be found in Dataverse: https://doi.org/10.34894/J0CM5K). Thus, just like other information retrieval CAs in other domains (Shawar and Atwell, 2007; Sumikawa et al., 2020), the CAVAA was able to answer (frequently asked) questions providing users with information that matches their needs. Finally, as can be seen in Figure 1, there was a third button users could click on to indicate that they did not desire any additional information. By clicking this button, users could proceed to indicate their opinion about the political attitude statement.

In addition to these buttons, the CAVAA had an open text field that could be used for asking open-ended questions about the statements, for example regarding the advantages or disadvantages of the topic at stake. By means of natural language understanding (NLU) methods, the user's utterance was parsed into predefined semantic slots and this way the CAVAA was able to provide the user a preformulated response. The CA was trained to recognize questions asking for the semantic meaning of terms in the statements, information with respect to the status quo, but also to provide a list of advantages or disadvantages of the policy discussed in the political statement.

A specific conversational flow was also created in case the bot was unable to recognize the user's question. When this situation occurred, the bot would first ask the user to reformulate the question. When the bot also failed to recognize the rephrased question, it was programmed to provide a list of topics the CAVAA did have answers to. This was done, because Følstad et al. (2018) have shown that it is important to be transparent about the functions and limitations of a CA. Furthermore, in order to engage users in the tool (Thies et al., 2017), the CAVAA started the conversation with: *Hello, good to hear that you are about to fill out a VAA!*, followed by: *Are you ready to start?*. Users were offered two buttons that said, *Yes, I'm ready!*, and *No, I'll come back another time*. This way, users were encouraged to actively participate in the conversation with the CAVAA from the start. Similar to the traditional VAA, users of the CAVAA also received a voting advice after answering the 20 statements.

Before the actual launch of our study, we conducted a pretest to check if (a) the CAVAA was well-programmed, (b) the additional background information actually made the statement more understandable, and (c) there was information missing in the explanations. Ten participants (4 males and 6 females, *M*_age_ 34.8 years, *SD* = 5.36) took part in the pretest. During the pretest the participants answered the 20 statements and interacted with the CAVAA. After each statement, the test leader asked questions about the semantic and pragmatic information available through the buttons. Moreover, the test leader monitored all of the interactions between the user and the bot. In this way the conversational flow could be checked. Based on the results of the pretest, several changes were made for the main study. For example, we rephrased some of our semantic explanations of terms in the questions, as we had first used new difficult words in our explanations.

### Participants

Data were collected online between 14 May 2020 and 1 June 2020. During this period a random link was actively promoted via Facebook and other social media platforms. The only requirement for participation was that respondents had to be eligible to vote. A total of 231 respondents completed the experiment. The data of two respondents were removed from the dataset, because they showed straight-lining behavior, providing the same answer to all questions about the dependent variables in this study.

Of the remaining 229 participants, 78 were male (34.1%), and 150 female (65.5%). One person answered that he/she would rather not say (0.4%). The average age was 30.43 years old (*SD* = 14.51), ranging from 18 to 75. Most of the participants received an education at University level, as they finished an undergraduate program (52.9%) or a master's program (26.2%). The remaining participants finished intermediate vocational education (12.7%) or high school (8.4%). Also, the majority of participants was familiar with VAAs (89.5%), and had previous experience with CAs (61.6%).

We used an aggregated index to distinguish participants into those with lower levels of political sophistication and those with higher levels of political sophistication. As explained earlier, the theoretical concept of political sophistication has a cognitive aspect (usually operationalized as educational level), an informative aspect (usually measured as general prior political knowledge), and a motivational component (usually measured as political interest; cf. Luskin, 1990; Rapeli, 2013; Stiers, 2016). We measured all these aspects in the pre-(CA)VAA survey.

First, political interest was measured with 3 items (e.g., *I am interested in politics*) on a 7-point Likert scale (1 = “completely disagree,” 7 = “completely agree”) based on Lachat (2008) and Shani et al. (2012). This scale showed to have a good reliability (α = 0.89, *M* = 4.78, *SD* = 1.53). Second, general prior political knowledge was measured with seven factual true-false statements about the Dutch political system (e.g., *There are 225 members in the House of Representatives*). Third, respondents were also asked to indicate their highest finished degree of education (on a seven-point scale). To construct one measure for political sophistication, we aggregated these three scores, which were all initially expressed on a seven-point scale, into a new 21-point scale variable. This aggregated score was subsequently used to create a high political sophistication group (*N* = 122, scores of 15.67 of higher) and a group with low levels of political sophistication (*N* = 107, scores of 15.33 or lower).

We compared respondents assigned to the VAA and CAVAA on several background variables to check for *a priori* differences between conditions. Respondents in the VAA and CAVAA condition were comparable with respect to gender [χ^2^(2) = 0.63; *p* = 0.73], whether or not they had used a VAA before [χ^2^(1) = 2.81; *p* = 0.09] and whether or not they had chatted to a CA before [χ^2^(1) = 0.79; *p* = 0.37]. The group of people who participated in the CAVAA condition was slightly younger on average, however [*t* (151.9) = 2.53; *p* = 0.01]. Because of this *a priori* difference, we ran all analyses both with and without age as a covariate.

### Measures and Procedure

The study started with an introductory text in which respondents were asked to provide informed consent for participation and data usage. When respondents consented, they were first asked some demographic questions. Next, they were randomly assigned to either the VAA or the CAVAA. After having answered the 20 attitude statements in either one of these conditions, users received a voting advice. Finally, they answered several attitude questions in a post-tool survey about the dependent variables: evaluation of the (CA)VAA experience, voting intention, perceived political knowledge and factual political knowledge.

People's evaluation of the (CA)VAA experience was measured on a seven-point scale with five items based on Heller et al. (2005) (e.g., *This tool was easy to use*). The items showed a good reliability (Cronbach's α = 0.87; *M* = 5.14, *SD* = 1.14).

Voting intention was measured by three items, two of which were based on Glynn et al. (2009) (i.e., *If there were elections now, I would vote* and *After consulting the (CA)VAA, I feel sufficiently informed to vote*) and one additional item that was constructed by the authors (i.e., *I plan to vote in the upcoming elections on March 17, 2021*). The “sufficiently informed” item from the original scale showed to load onto a different construct in a factor analysis and this item also decreased the reliability of the scale. Therefore, it was decided to include only the other two items (α = 0.93, *M* = 6.35, *SD* = 0.99).

Perceived political knowledge was measured on a seven-point scale with three items based on Ladner (2012) (e.g., *By using (CA)VAAs, I have gained more understanding of the political landscape)*. The scale showed to have a good reliability (α = 0.79, *M* = 4.28, *SD* = 1.35).

Factual political knowledge was measured with six open-ended knowledge questions related specifically to the political statements in the (CA)VAA (i.e., *What is a binding referendum?* and *What is the current state of affairs regarding the retirement age?*). The answers were scored based on a coding scheme (the coding scheme is available in Dataverse: https://doi.org/10.34894/J0CM5K). To check the reliability of the ratings, a random set of 120 answers^4^ was coded by two independent coders. The interrater reliability of the coding was found to be κ = 0.85 (*p* < 0.001), which indicates an almost perfect agreement based on interpretation of Cohen's Kappa statistic for strength of agreement (Landis and Koch, 1977; Viera and Garrett, 2005).

### Results

#### (CA)VAA Experience

For evaluation of the (CA)VAA experience, results showed a significant main effect of VAA type [*F* (1, 225) = 19.56, *p* < 0.001, Cohen's *d* = 0.64]. As can be read from Table 1, the evaluation of the CAVAA experience was more positive compared to the experience of the regular VAA. There was no main effect of political sophistication [*F* (1, 225) = 0.04, *p* = 0.84], and also the interaction failed to reach significance [*F* (1, 225) = 0.07, *p* = 0.79].

**Table 1:** Means and Standard Deviations of the (CA)VAA experience (ranging from 1 – a low evaluation to 7 – a high evaluation) in each condition.

**Type of tool**	**Political sophistication**	***M (SD)***	***N***
VAA	Low	4.73 (1.18)	37
	High	4.72 (1.06)	51
	Total	4.72 (1.11)	88
CAVAA	Low	5.36 (0.93)	70
	High	5.43 (1.23)	71
	Total	5.39 (1.09)	141

#### Voting Intention

In contrast to what we have seen for (CA)VAA experience, analyses for voting intention showed no significant main effect of VAA type [*F* (1, 225) = 3.28, *p* = 0.07]. Results did reveal a main effect of political sophistication [*F* (1, 225) = 30.23, *p* < 0.001, Cohen's *d* = 0.79]: those with higher levels of political sophistication report to be more likely to vote than those with lower levels of political sophistication (see Table 2). There was no significant interaction effect between VAA type and political sophistication [*F* (1, 225) = 2.00, *p* = 0.16].

**Table 2:** Means and Standard Deviations of voting intention (ranging from 1 – a low voting intention to 7 – a high voting intention) in each condition.

	**Political sophistication**	***M (SD)***	***N***
VAA	Low sophistication	6.22 (0.83)	37
	High sophistication	6.73 (0.50)	51
	Total	6.51 (0.70)	88
CAVAA	Low sophistication	5.81 (1.30)	70
	High sophistication	6.68 (0.69)	71	
	Total	6.25 (1.12)	141

#### Perceived Political Knowledge

For perceived knowledge, analyses showed a significant main effect of VAA type [*F* (2, 225) = 13.98, *p* < 0.001, Cohen's *d* = 0.60]: a respondent's perceived political knowledge is higher after having worked with a CAVAA (see Table 3). There was no significant main effect of political sophistication [*F* (1, 225) = 0.001, *p* = 0.98], nor an interaction effect between the type of VAA and political sophistication [*F* (1, 225) = 0.15, *p* = 0.70]. Hence, the effect of VAA type (traditional VAA vs. CAVAA) does not depend on one's level of political sophistication.

**Table 3:** Means and Standard Deviations of perceived political knowledge score (ranging from 1 – a low perceived knowledge score to 7 – a high perceived knowledge score) in each condition.

	**Political sophistication**	***M (SD)***	***N***
VAA	Low sophistication	3.90 (1.21)	37	
	High sophistication	3.84 (1.46)	51
	Total	3.86 (1.35)	88
CAVAA	Low sophistication	4.50 (1.12)	70
	High sophistication	4.58 (1.38)	71
	Total	4.54 (1.28)	141

#### Factual Political Knowledge

With regard to people's objective political knowledge, analyses again showed a significant main effect of VAA type [*F* (1, 225) = 6.68, *p* = 0.01, Cohen's *d* = 0.37]: after having worked with a CAVAA respondents were better able to answer the factual knowledge questions about the political issues addressed in the tool (see Table 4). There was also a significant main effect of political sophistication [*F* (1, 225) = 15.06, *p* < 0.001, Cohen's *d* = 0.60]: people with higher levels of political sophistication were better in answering the knowledge questions. Just as for perceived political knowledge, no interaction effect was found between type of VAA and political sophistication [*F* (1, 225) = 0.05, *p* = 0.81].

**Table 4:** Means and Standard Deviations of the number of factual political knowledge questions answered correctly (Min. = 0; Max. = 6) in each condition.

	**Political sophistication**	***M (SD)***	***N***
VAA	Low sophistication	0.76 (0.93)	37
	High sophistication	1.53 (1.38)	51
	Total	1.20 (1.26)	88
CAVAA	Low sophistication	1.29 (1.45)	70
	High sophistication	1.97 (1.48)	71
	Total	1.63 (1.50)	141

### Conclusion and Discussion Study 1

Study 1 showed CAVAAs outperform traditional VAAs on a number of points: users evaluate their experience more positively, and both the perceived and factual political knowledge scores were higher. These findings confirm our expectations; a CAVAA can function well as a task-oriented information seeking system that helps users to achieve their goal in this specific usage context, i.e., obtaining political knowledge against low cognitive costs.

Counter to expectations, however, we did not observe a difference between the two types of tools for the intention to cast a vote. This is strange in light of earlier research showing a strong link between political knowledge and vote choice (Delli Carpini and Keeter, 1996). One explanation for the lack of effects for voting intention might be that when conducting the experiment the next national elections were scheduled to take place in 9 months (on March 17, 2021). As 9 months might have felt too far away for participants, they may have just responded in a socially desirable way (see also Traugott and Katosh, 1979), also explaining the very high voting intention scores across the board (see Table 2).

As the positive results of CAVAAs on users' perceived and factual political knowledge and tool experience are promising, we conducted a second experimental study to gain a better understanding of the CAs' design principles that match the users' needs best. The CAVAA used in Study 1 contained a semi-structured design, in which users could both click on pre-defined buttons and type in their questions in an open chat window. In Study 2 we tested this semi-structured design against a fully structured design that only contains information buttons and to a non-structured design with only an open chat function.

Similar to Study 1, we examined the effects of these three CAVAA designs on users' perceived and factual political knowledge. For these variables we expect that, because users are in a mode of low elaboration (Kamoen and Holleman, 2017), a structured CAVAA design may be most beneficial as this design requires the lowest effort; people just have to click a button to access relevant information. This expectation matches with research on CAs in other domains, showing efficiency effects for structured designs relative to open chatbot designs (Jain et al., 2018). Moreover, as lower educated users have been shown to experience difficulties in phrasing questions themselves (Elling et al., 2012), we expect that a structured design leads to higher political knowledge scores for users with lower levels of political sophistication in particular.

Besides perceived and factual political knowledge, we also examined the effect of the three designs on tool evaluation. Compared to Study 1 we refined this measurement by focusing on three well-known aspects of tool evaluation: perceived usefulness, perceived ease of use, and perceived playfulness (Van der Heijden, 2003; Castañeda et al., 2007; Lee and Chang, 2011). The first two of these concepts are derived from the Technology Acceptance Model (TAM) that describes how people come to accept and use technology (see Davis, 1989, and more recent elaborations of that model by Ahn et al., 2007). Perceived usefulness has been defined as people's desire to benefit from the technology in terms of productivity or job performance, whereas perceived ease of use addresses the efficiency of a tool in terms of the degree to which users experience using the tool is simple and free of effort (Davis, 1989). In addition to these two factors from the TAM model, we also measured perceived playfulness which has been defined as people's enjoyment in using technology (Ahn et al., 2007) to cover the entertainment motivation of VAA users (Van de Pol et al., 2014).

Research on CAs in other domains has demonstrated that the usage of buttons in a structured design make a CA easy to use (Jain et al., 2018). At the same time, buttons remove the limitations, such as miscommunication, that might play a role in a non-structured chatbot interface (Klopfenstein et al., 2017). Therefore, we expect the structured design to be easier to use than the non-structured design. This effect is probably larger for users with lower levels of political sophistication as they require more structure. Moreover, Liao et al. (2020) have found that a structured design leads users to the path of actually clicking on the buttons. We therefore also expect that users will actually access more information and evaluate the structured design to be more useful. This probably also counts to a larger extent for users with lower levels of political sophistication who may experience difficulties recognizing their own comprehension problems and formulating a self-phrased question about it (Elling et al., 2012).

As for perceived playfulness, two scenarios are possible. On the one hand, it is known that the entertainment value of a chatbot is high if the bot meets the expectations of its users and people generally expect that they can ask questions themselves (Hedberg, 1998). On the other hand, VAA users are known to be in a processing mode of low elaboration and therefore they may simply not like to put in the effort of typing a question in an open chat window. In Study 2 we will explore the effect of the three designs and their interaction with the user's level of political sophistication for perceived playfulness.

## Study 2: Method

### Design

An experiment was set up with a 3 (CAVAA design: structured, semi-structured, or non-structured) x 2 (Political sophistication: low or high) between-subjects design. Respondents were randomly assigned to one of the three CAVAA conditions, political sophistication was measured as a quasi-experimental variable. The Research Ethics and Data Management Committee of Tilburg University granted permission to conduct the study (reference number: REDC #2020/061).

### Materials and Procedure

**Figure 2:**
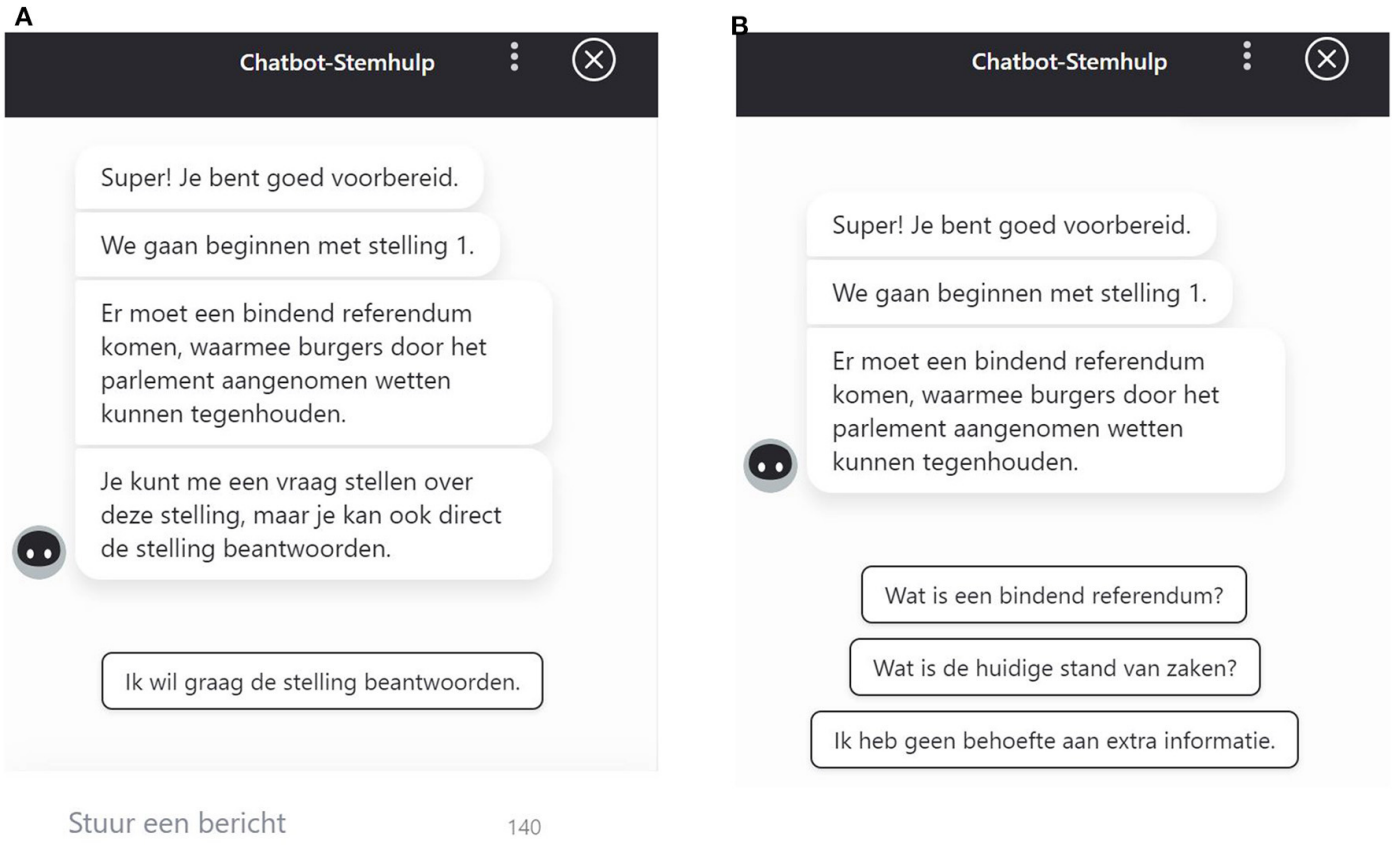
**(A)** Example of the non-structured CAVAA design (*We will start with statement 1. There should be a binding referendum with which citizens can stop laws being implemented*. *You can ask me a question about this statement or directly respond to the statement*) showing a button to directly answer the question (*I want to answer the question*) and an open text field. **(B)** Example of the structured CAVAA design (*We will start with statement 1. There should be a binding referendum with which citizens can stop laws being implemented*. *You can ask me a question about this statement or directly respond to the statement*) with buttons that can be used for accessing additional information (*What is a binding referendum, What is the current state of affairs*, and *I do not need additional information*).

For this second study, we used the CAVAA constructed for our first study as a starting point, and created two new versions using the Flow.ai software: one version without buttons where participants only had the opportunity to ask the CAVAA an open question using the chat field (non-structured design), and another version that contained only buttons and no text field (structured design, see Figures 2A,B for examples). Similar to our first study, respondents could indicate their answer to the CAVAA statements on a three-point scale (agree-neutral-disagree), or choose a no-opinion answer. After respondents had answered all 20 statements in the CAVAA, they were given a voting advice and were asked to fill in questions about the dependent variables.

### Participants

We recruited a convenience sample of participants by distributing the link to our study via various social media channels. The study was accessible between 14 May and 1 June 2020 and yielded 180 respondents who completed the experiment. We checked the answers of these respondents for straight-lining behavior, but none of the participants gave the same answer to all attitude questions.

Of the 180 participants, 77 were male (42.8%) and 103 female (57.2%). Age ranged between 17 and 85 years, with a mean age of 35.7 (*SD* = 19.86). A total of 19 respondents (10.7%) had received an education at senior secondary vocational education level (i.e., MBO level) or lower than that; 155 participants (86%) completed or were currently enrolled in higher educated (higher vocational, i.e., HBO or university level), and the remaining 6 participants reported “other” as educational level but failed to specify their education in the open-ended follow-up question (3.3%). 85.6% of the participants had filled out a VAA before, and 66.7% had experience with using CAs.

We checked the distribution of respondents across the three CAVAA conditions for gender [χ^2^(2) = 1.83, *p* = 0.400], age [*F* (2, 177) = 0.63, *p* = 0.537], educational level [χ^2^(12) = 14.41, *p* = 0.273], prior chatbot experience [χ^2^(2) = 0.05, *p* = 0.997] and prior VAA experience [χ^2^(2) = 0.78, *p* = 0.677], and no differences were observed. This means that there is no reason to assume that there were *a priori* differences between the respondents in the CAVAA conditions.

Just like in Study 1, we measured the respondents' educational level (on a seven-point scale), political interest (3 items based on Shani et al., 2012; α = 0.88), and general prior political knowledge (7 true-false questions based on Stiers, 2016) and created an additive index out of these three measures indicating level of political sophistication. Based on this index value, we created two approximately equal groups of respondents: those with an aggregated index up until 15.33 were classified as respondents with lower levels of political sophistication (*N* = 88; 48.9%), and those respondents with higher scores were classified to have higher levels of political sophistication (*N* = 92; 51.1%).

### Measures

Similar to Study 1, perceived political knowledge was measured with three items to be answered on a 7-point Likert scale ranging from 1 (“strongly disagree”) to 7 (“strongly agree”). The items were based on Ladner (2012). An example item is *By using this chatbot, I have the feeling I understand the political issues better* (*M* = 4.36, *SD* = 1.27, α = 0.65). We again also measured the respondent's factual political knowledge by six open-ended knowledge questions about topics that were addressed in the CAVAA. As these were the same questions as those used in Study 1, we also applied the same coding scheme for classifying the statements (the full coding scheme can be found in Dataverse: https://doi.org/10.34894/J0CM5K).

Compared to Study 1, we refined the measurement of the evaluation of the CAVAA experience, by focusing on three strong predictors of users' attitude toward technology and adopted the measures of Ahn et al. (2007). Perceived usefulness was measured with five questions. An example question is *It is more efficient to use a CAVAA than a regular VAA*. The reliability of the scale was good (α = 0.89, *M* = 5.27, *SD* = 1.20). Perceived ease of use was also measured with five items, such as *It was easy to work with the CAVAA*. The items also showed a high internal consistency (α = 0.74, *M* = 5.81, *SD* = 0.85). Lastly, five items were included to measure perceived playfulness. An example is the statement *It was fun to interact with this Voting Advice Application*. Again, the reliability of the scale was high (α = 0.87, *M* = 5.25, *SD* = 1.07).

### Results

#### Perceived and Factual Political Knowledge

The mean perceived and factual knowledge scores and their standard deviations are indicated in Table 5. A two-way ANOVA revealed that the perceived knowledge does not depend on the CAVAA version [*F* (2, 174) = 1.60; *p* = 0.205], nor on the level of political sophistication [*F* (1, 174) = 3.50; *p* = 0.063], or on the interaction between the two [*F* (2, 174) = 0.26; *p* = 0.773]. The same pattern was found for factual knowledge, where there was also no difference observed between the three CAVAA conditions [*F* (2, 174) = 2.96; *p* = 0.055], the different levels of political sophistication [*F* (1, 174) = 2.39; *p* = 0.124], and also the interaction between these variables failed to reach significance [*F* (2, 174) = 0.59; *p* = 0.556].

**Table 5:** Means and Standard Deviations of perceived knowledge (ranging from 1 - a low score to 7 - high score) and factual knowledge (ranging from 0 - a low score to 6 - a high score) in each condition.

**CAVAA**	**Sophistication**	**Perceived knowledge *M (SD)***	***N***	**Factual knowledge *M (SD)***	***N***
Non-structured	Low	4.60 (1.23)	19	1.74 (1.24)	19
	High	4.03 (1.41)	37	2.20 (1.37)	35
	Total	4.22 (1.37)	56	2.04 (1.33)	54
Semi-structured	Low	4.75 (1.16)	33	2.61 (1.77)	33
	High	4.45 (1.19)	35	2.63 (1.21)	35
	Total	4.59 (1.18)	68	2.62 (1.50)	68
Structured	Low	4.31 (1.17)	36	2.06 (1.43)	36
	High	4.08 (1.45)	20	2.60 (1.39)	20
	Total	4.23 (1.27)	56	2.25 (1.43)	56

#### CAVAA Experience

The means and standard deviations for perceived ease of use, perceived usefulness and perceived playfulness can be found in Table 6.

**Table 6:** Means and Standard Deviations of the different aspects of CAVAA experience (ranging from 1 – a low evaluation to 7 – a high evaluation) in each condition.

**CAVAA**	**Sophistication**	**Usefulness *M (SD)***	***N***	**Ease of use *M (SD)***	***N***	**Playfulness *M (SD)***	***N***
Non-structured	Low	6.12 (0.60)	19	6.13 (0.62)	19	5.56 (0.43)	19
	High	5.24 (1.09)	37	5.94 (0.66)	37	5.30 (0.75)	37
	Total	5.54 (1.04)	56	6.00 (0.64)	56	5.39 (0.67)	56
Semi- structured	Low	5.42 (1.07)	33	5.94 (0.89)	33	5.46 (1.07)	33
	High	5.21 (1.49)	35	5.78 (0.84)	35	5.34 (1.25)	35
	Total	5.31 (1.29)	68	5.86 (0.86)	68	5.40 (1.16)	68
Structured	Low	4.96 (1.29)	36	5.57 (1.06)	36	4.87 (1.35)	36
	High	4.93 (0.99)	20	5.57 (0.84)	20	5.03 (0.98)	20
	Total	4.95 (1.18)	56	5.57 (0.98)	56	4.93 (1.23)	56

For perceived usefulness, we observed a main effect of CAVAA condition [*F* (2, 174) = 5.00; *p* = 0.008]. A *post-hoc* test (Bonferroni) revealed the structured CAVAA version was evaluated to be more useful than the non-structured version (*p* = 0.025), while there were no differences between the other CAVAA types (structured vs. semi-structured: *p* = 0.552; semi-structured vs. non-structured: *p* = 0.239). We also observed a main effect of political sophistication [*F* (1, 174) = 4.21; *p* = 0.042], such that the lower sophisticated group, on average, evaluated the different CAVAAs to be more useful as compared to their higher sophisticated peers. Finally, the interaction between CAVAA type and political sophistication failed to reach significance [*F* (2, 174) = 1.85; *p* = 0.16].

The pattern of results for our second indicator of CAVAA experience, ease of use, was comparable to a large extent. Here, we also observed a main effect of CAVAA version [*F* (2, 174) = 3.91; *p* = 0.022]. Similar to the results for usefulness, the structured version was evaluated better than the non-structured version (*p* = 0.023), whereas there were no differences observed between the other CAVAA conditions (structured vs. semi-structured: *p* = 1; semi-structured vs. non-structured: *p* = 0.182). In contrast to the results for usefulness, we did not observe a main effect of political sophistication this time [*F* (1, 174) = 0.79; *p* = 0.374], and also the interaction between CAVAA version and political sophistication failed to reach significance [*F* (2, 174) = 0.20; *p* = 0.817].

Lastly, perceived playfulness also displayed a pattern of results largely comparable to the other indicators of CAVAA experience. For this factor we observed a main effect of CAVAA version [*F* (2, 174) = 3.42; *p* = 0.035], such that there was a tendency for the structured version to be evaluated more playful than the non-structured version (*p* = 0.068) and that the semi-structured version was evaluated to be more playful than the non-structured version as well (*p* = 0.044). The main effect of political sophistication did not reach significance [*F* (1, 174) = 0.19; *p* = 0.663], nor did the interaction between CAVAA version and sophistication [*F* (2, 174) = 0.50; *p* = 0.605].

#### Qualitative Exploration of the CAVAA Conversations

As we found a preference for the structured design over the non-structured design on all of the experience measures, a relevant question is as to what is the underlying cause of this preference. For one, the non-structured design may be evaluated less positively, because users had to make too much of an effort asking their question to the tool and hence that this design still does not offer low-key enough options for accessing additional information. Alternatively, it could be the case that users were triggered to ask their questions, but that they experienced miscommunication with the non-structured version of the tool, leading to more break-offs.

To explore which of these two explanations is most likely, we randomly selected 30 chatbot conversations for each of the 3 CAVAA versions, with in each conversation 20 statements. This led to 30 × 20 × 3 = 1,800 instances of a respondent responding to a political attitude statement. For all of these instances, we coded (a) whether or not the respondent actually answered all 20 statements or left the CAVAA early (b) whether semantic information was requested and (c) whether pragmatic information was requested. A subset of 18 conversations (with 20 statements each unless the conversations ended early) were coded by two independent coders and their agreement was high (Kappa between 0.93 and 1).

Table 7 shows that twenty conversations of the total sample of ninety were ended early. To test if the break-off rate differs per version, a one-way ANOVA was performed. The analysis shows no significant difference between CAVAA versions on the number of conversations ended early [*F* (2, 87) = 0.82, *p* = 0.44].

**Table 7:** Task completion per CAVAA design.

	**The number of conversations ended** **early (%)**
Structured	5 (16.67%)
Semi-structured	9 (30.00%)
Non-structured	6 (20.00%)
Total	20 (22.22%)

Table 8 shows how frequently information was requested to the three CAVAA versions. For presentational clarity, it was decided to separately report for the semi-structured version how many information requests were handled through the buttons, and how many via asking open questions in the chat window.

**Table 8:** The total number of semantic and pragmatic information requests per CAVAA design.

	**Semantic requests**	**Pragmatic requests**	**Total**	
Structured	92	120	212	
Semi-structured (Buttons)	112	128	240	
Semi-structured (Chat)	2	-	2	
Non-structured	61	29	90	
Total	267	277	544	

A first thing that stands out when looking at Table 8 is that if the chatbot offers both buttons and an open chat field, the latter is hardly used for obtaining information. In the semi-structured design only two open questions were asked via the open chat function; all other information requests in the semi-structured version are handled through the buttons. This gives an indication that the buttons are useful for users.

As for the total number semantic information requests, a cross-classified multi-level model indicates that there is a difference between versions such that in the semi-structured version more requests for semantic information were filed relative to both the structured [χ ^2^(1) = 5.33; *p* < 0.001] and the non-structured version [χ^2^(1) = 24.06; *p* < 0.001]. There is also a difference between the structured and the non-structured version such that more semantic requests were done in the structured than in the non-structured version [χ^2^(1) = 7.51, *p* < 0.001].

Also, the chance that a pragmatic information request is made depends on the CAVAA version. There is a higher chance that a respondent requests pragmatic information in the structured or semi-structured version as compared to the non-structured version [χ^2^(1) = 16.83; *p* < 0.001 and χ^2^(1) = 9.83; *p* < 0.001 respectively], whereas no difference is found between the structured and the semi-structured version [χ^2^(1) = 0.14; *p* = 0.71]. All in all, these results suggest that buttons (in the structured and the semi-structured version) trigger respondents to ask for additional information.

## Overall Discussion

### VAAs vs. CAVAAs

Study 1 shows that a CAVAA outperforms a traditional VAA in different ways: CAVAA users report higher levels of perceived political knowledge, they answer more factual knowledge questions correctly, and their tool experience is evaluated more positively. These effects apply both to users with high levels of political sophistication and to users with low levels of political sophistication. Integrating a VAA into a CA is thus a promising way to support users in their question comprehension process. This is in line with findings by Galesic et al. (2008), who show that the lesser effort respondents have to make to access additional information, the more likely they are to do so. The current study adds that providing additional information that users can easily request in a real-time tool, at least in this specific context, does not only result in a better performance on perceived and factual knowledge measures, but that it also leads to more positive evaluations.

A point of discussion with respect to Study 1 concerns the question as to what has actually caused the CAVAA to outperform the traditional VAA on knowledge and experience measures. Not only did the way of information presentation differ between conditions (static website vs. a more dynamic CA lay-out), also the amount of information presented differed, as the CAVAA did contain additional information and the VAA did not. This choice was made in eye of the ecological validity, as a great majority of current VAAs do not offer any additional information. However, the repercussion of this choice is that we now are uncertain as to what has caused the differences between VAAs and CAVAAs: the information presentation in a CA lay-out, or additional information as such. To examine which of these two factors was the key determinant of the more positive evaluations, a future study should compare a traditional VAA to both a VAA with additional information and a CAVAA with the same information presented in a more dynamic way.

### CAVAA Design

Study 2 shows for both users with higher and lower levels of political sophistication that, while the design of the tool does not affect the perceived and factual knowledge scores, it does affect the perception of usefulness, ease of use, and playfulness. In all of these cases, and in line with expectations based on the literature, a structured design with buttons is preferred to a non-structured design with an open text field. A qualitative exploration of these results indicates more requests for semantic and pragmatic information were filed in the versions that contained buttons, whereas the number of break-offs did not differ between the three designs. This suggests that the structured design responds to the processing mode of low elaboration best, rather than being evaluated best because of miscommunication issues playing a role in the non-structured design. This finding matches with research on CAs in other domains, showing efficiency effects for structured designs relative to open chatbot designs (Jain et al., 2018).

A point for future research is to investigate if there are other ways of information presentation that could even further enhance the CAVAA experience and perhaps also the political knowledge gained from the tool. Compared to a text-based CA, for example, a voice-based CA is not only a much faster tool, but it can also be more appealing and enhance understanding of complex topics, such as political issues, as voice can elicit emotions like enjoyment that stimulate learning effects (Angga et al., 2015; Hernik and Jaworska, 2018; Obergriesser and Stoeger, 2020). Future research could therefore compare a text-based and a voice-based CA not only on political knowledge and tool evaluation measures, but also on the amount and type of information CAVAA users request by analyzing the chatlogs. We expect users of voice-based chatbots to request more political information and experience more enjoyment.

### Political Knowledge

In both studies we measured the respondent's perceived and factual political knowledge. In our view, measuring these two factors is important, because research has shown that perceived and factual measures do not always correlate highly (Kamoen et al., 2015). Moreover, both factors are important in the current political context; one's political self-confidence is one of the main factors explaining turn-out (Delli Carpini and Keeter, 1996) and the more factual knowledge one possess the more closely the vote casted probably matches the actual underlying opinion of a respondent.

Although we did find beneficial effects of a CAVAA over a VAA in obtaining (perceived and factual) political knowledge in Study 1, it is striking that no differences were found between the three CAVAA designs in Study 2. The qualitative content analysis, however, showed that users request more information by means of buttons than by typing in questions via the open text field. Therefore, one may have expected that those who worked with a structured CAVAA with buttons also indicated to have obtained more political knowledge since they clearly requested more information. The mere facts that we could not demonstrate a difference for our political knowledge indicators may be due to the fact that political knowledge was only measured after using the CAVAA; perhaps if one were to measure knowledge both before and after the experiment a delta score of the knowledge difference is a more sensitive measure for demonstrating differences between versions.

Another point of discussion related to the measurement of political knowledge, concerns the positioning of these measurements within both of our experiments. In order to avoid the factual knowledge items to affect measurement of the other outcome variables, we decided to place the knowledge items at the very end of the survey in both of our studies. This, however, resulted in some blank answers. As it was possible for respondents to leave the open ended factual knowledge questions blank, we cannot be sure if no response indicates that the respondent did not know the answer, or instead, that the respondent was just too tired to answer. In a future study it would therefore be better to either reconsider to positioning of factual knowledge questions within the post-tool survey, or to use a different type of response format for these questions. Delli Carpini and Keeter (1993) and Dolan (2011) suggest to use a closed ended response format for measuring political knowledge in which respondents are presented with statements about politics and have to indicate whether this statement is true, false, or whether the respondent does not know. Another advantage of this type of measurement is that there are not multiple coders needed for categorizing the answers provided.

### Political Sophistication

We observed several main effects of political sophistication showing that the higher sophisticated users score higher on factual knowledge and on voting intention (Study 1) and that the lower sophisticated users evaluate all three CAVAA designs to be more useful than the higher sophisticated users (Study 2). These main effects are largely in line with what one would expect for higher and lower sophisticated users. Contrary to expectations, however, we were never able to show interactions between the level of political sophistication and the type of tool in Study 1 (VAA or CAVAA) or the type of CAVAA design in Study 2 (structured, semi-structured, or non-structured).

In the literature there is a lot of discussion about how the concept of political sophistication can best be measured (Luskin, 1990). In contrast to the many previous studies (e.g., Macdonald et al., 1995; Gomez and Wilson, 2001), we decided to measure all three aspects relevant for the concept of political sophistication: political interest, educational level, and political knowledge (Luskin, 1990; Rapeli, 2013; Stiers, 2016). At a first sight the type of measurement is therefore unlikely to be the cause of the unexpected results, as its construct validity was high relative to measurements applied in previous studies.

A more plausible explanation for the lack of interaction effects is the composition of the sample. With respect to perhaps the most important ingredient on the user's political sophistication, educational level, there was only little variation in the sample of both Study 1 (80% of respondents received a high level of education) and Study 2. It is therefore recommendable to replicate the studies reported here with a more diverse sample of respondents with respect to the level of education.

### Practical Implications

Task-oriented conversational agents (CAs) are used in many different domains, but until now they had not been applied in the context of political VAAs. The current study shows that it is recommendable to implement VAAs in a Conversational Agent, as this leads to a better evaluation and higher (perceived and factual) knowledge scores as compared to a traditional VAA. As for the specific CAVAA design, we recommend developers to use a structured set-up with buttons rather than a non-structured design with only an open text field. This design is to be preferred in this specific usage context, and arguably also in other context where users are in a processing mode of low elaboration, because the buttons give hint about what kind of information can be accessed, and this information can be accessed in just one mouse click. We hope that CA and VAA designers further experiment with using CAs in this context, as CAVAAs are a promising way to decrease comprehension problems in a political attitude context, and probably also in other domains where users have to process difficult information and want to make only a very limited effort to do so.

## Conclusion

In election times, millions of voters consult Voting Advice Applications (VAAs) to learn more about political parties and their standpoints. While these tools positively affect voters' political knowledge, research also demonstrates that users experience comprehension problems when answering the VAA statements (Kamoen and Holleman, 2017). Moreover, users make a limited effort to resolve these comprehension problems. Our research is the first to implement a Conversational Agent (CA) into a Voting Advice Application. In a CAVAA, voters can request additional political information when responding to VAA statements. Our study shows that, relative to a regular VAA, users evaluate a CAVAA more positively and obtain more political knowledge. As for the specific design, a structured CAVAA with buttons receives better evaluations than a non-structured design with an open chat functionality. This is because the structured design better responds to the user's processing mode of low elaboration, showing from an increased number of information requests made. Our research thereby contributes to both the field of CA research and the field of experimental psychology, demonstrating the added value of CAs in the specific and important context of political VAAs where users make only a very minimal effort; CAs can trigger users to elaborate at least a little bit more.

## Data Availability Statement

The datasets presented in this study can be found in online repositories. The data have been archived in Dataverse; (Kamoen and Liebrecht, 2021).

## Ethics Statement

The studies involving human participants were reviewed and approved by the Research Ethics and Data Management Committee of our university, identification code: REDC #2020/060 (for study 1) and #2020/061 (for study 2). The participants provided their written informed consent to participate in this study.

## Author Contributions

Both authors listed have made a substantial, direct, and intellectual contribution to the work and approved it for publication.

## Conflict of Interest

The authors declare that the research was conducted in the absence of any commercial or financial relationships that could be construed as a potential conflict of interest.

## Publisher's Note

All claims expressed in this article are solely those of the authors and do not necessarily represent those of their affiliated organizations, or those of the publisher, the editors and the reviewers. Any product that may be evaluated in this article, or claim that may be made by its manufacturer, is not guaranteed or endorsed by the publisher.

## Footnotes

^1^The design of this first study actually contained two versions of a CAVAA: one in which the bot talked to the respondent with a personal and engaging communication style (i.e., conversational human voice; Kelleher and Miller, 2006) that contained elements of personalization (e.g., a personal greeting, *Hi name*), informal language (e.g., emoticons, ) and invitational rhetoric (e.g., showing sympathy, *enjoy!*), and one with a formal tone of voice in which these elements were absent. As no differences between these two bot conditions were observed for any of the DVs in our study, we decided to merge the two conditions together for presentational clarity. In the Master thesis of Van Limpt (2020) that forms the basis of Study 1, the reader can access the full analyses.

^2^ www.qualtrics.com

^3^ www.flow.ai

^4^As we will explain later on, the same set of knowledge questions was used in Study 1 and Study 2. The set of 120 answers that was analyzed by both coders concerned a random set of the combined set of answers from Study 1 and Study 2.
